# Potential Antioxidant Role of Tridham in Managing Oxidative Stress against Aflatoxin-B_1_-Induced Experimental Hepatocellular Carcinoma

**DOI:** 10.1155/2012/428373

**Published:** 2012-02-01

**Authors:** Vijaya Ravinayagam, Ravindran Jaganathan, Sachdanandam Panchanadham, Shanthi Palanivelu

**Affiliations:** ^1^Department of Pathology, Dr. ALM Post-Graduate Institute of Basic Medical Sciences, University of Madras, Taramani Campus, Tamil Nadu, Chennai 600113, India; ^2^Department of Medical Biochemistry, Dr. ALM Post-Graduate Institute of Basic Medical Sciences, University of Madras, Taramani Campus, Tamil Nadu, Chennai 600113, India

## Abstract

Hepatocellular carcinoma (HCC) is one of the most fatal cancers due to delayed diagnosis and lack of effective treatment options. Significant exposure to Aflatoxin B_1_ (AFB_1_), a potent hepatotoxic and hepatocarcinogenic mycotoxin, plays a major role in liver carcinogenesis through oxidative tissue damage and p53 mutation. The present study emphasizes the anticarcinogenic effect of Tridham (TD), a polyherbal traditional medicine, on AFB_1_-induced HCC in male Wistar rats. AFB_1_-administered HCC-bearing rats (Group II) showed increased levels of lipid peroxides (LPOs), thiobarbituric acid substances (TBARs), and protein carbonyls (PCOs) and decreased levels of enzymic and nonenzymic antioxidants when compared to control animals (Group I). Administration of TD orally (300 mg/kg body weight/day) for 45 days to HCC-bearing animals (Group III) significantly reduced the tissue damage accompanied by restoration of the levels of antioxidants. Histological observation confirmed the induction of tumour in Group II animals and complete regression of tumour in Group III animals. This study highlights the potent antioxidant properties of TD which contribute to its therapeutic effect in AFB_1_-induced HCC in rats.

## 1. Introduction

Hepatocellular carcinoma (HCC) is the most common type of primary liver cancer and accounts for around 70% of all liver cancers [1]. Various factors have been implicated as risk factors in the pathogenesis of liver cancer, notably food contaminated with aflatoxins, toxins produced by fungi of the genus *Aspergillus* sp. (*A. flavus and A. parasiticus*) [2, 3]. However, oxidative stress has emerged as a key player in the development and the progression of liver cirrhosis [4] which is known to be a precursor of HCC. Both hepatitis B virus (HBV) and hepatitis C virus (HCV) appear to be particularly more potent in inducing oxidative stress, suggesting unique mechanisms that are activated by these viral infections. AFB_1_ acts as a strong hepatotoxicant, mutagen and naturally occurring hepatocarcinogen, which cause liver cancer in a dose-dependent manner [5]. AFB_1_ is metabolized by cytochrome P_450_ enzymes to aflatoxin-8,9 epoxide which is then detoxified by the glutathione S- transferase system (GST). This reactive metabolite escapes from the detoxification process and usually conjugates with DNA nucleotides forming adducts [6]. Such adducts are responsible for the generation of observable AFB_1_ inducible lethal mutation. AFB_1_ induces lipid peroxidation in rat liver, and this may be an underlying mechanism of carcinogenesis [7]. G to T transversion mutations in codon 249 of the p53 tumour-suppressor gene have been found in human liver tumour from geographic areas with high risk of aflatoxin exposure and in AFB_1_-induced liver toxicity [8].

Free radicals can be defined as molecules or molecular fragments containing one or more unpaired electrons in atomic or molecular orbitals formed during a variety of biochemical reactions and cellular functions. Reactive oxygen species (ROS) are free radicals of reactive anions formed by the incomplete one-electron reduction of oxygen including singlet oxygen; superoxides; peroxides; hydroxyl radicals [9]. ROS have been incriminated in the pathophysiology of a large number of diseases including coronary heart disease, neurodegenerative disorders like Alzheimer's disease, ageing, and cancer [10–12]. Oxidative damage/stress results when the level of ROS overpowers the system's ability to neutralize and eliminate them. Increased level of ROS usually results from lack or functional disturbance in antioxidant molecules or due to overproduction of ROS from the surrounding environment [13]. Recent studies show that ROS also have a role in cell signaling, including apoptosis, gene expression and the activation of cell signaling cascades [14].

Free radicals can cause damage in structural and metabolic components like lipids, proteins, enzymes, carbohydrates and DNA in cells and tissues. This ultimately results in membrane damage, fragmentation, or random cross-linking of molecules like DNA, enzymes, and structural proteins and even lead to cell death induced by DNA fragmentation, lipid peroxidation and finally to cancer formation [15]. Aerobic organisms have well-developed mechanisms to efficiently neutralize the oxidative effects of oxygen and its reactive metabolites. These self-sustained protective components are classified as the “antioxidant defense system” The sensitive balance between prooxidant and antioxidant forces in the body appears to be crucial in determining the state of health and well-being [16]. Under normal conditions, there is a balance between both the activities and the intracellular levels of these antioxidants. This balance is essential for the survival of organisms and their health [17]. Cumulative effect of the antioxidant defense system effectively removes the excess levels of prooxidants keeping the pro- to antioxidant ratio in an equilibrium state. Any loss in the functional activity of the major antioxidants leads to disruption in the prooxidant to antioxidant ratio, creating oxidative stress, and then cell damage ultimately favoring the process of carcinogenesis [18]. When the antioxidant system fails to counteract the increased productivity of ROS during pathological conditions, it results in oxidative stress and this is a preliminary event in the cancer initiation [13]. Supplementation of antioxidant rich diet is often recommended as part of cancer prevention [19]. There is a well-documented association between increased consumption of antioxidants and decreased incidence of cancer [20].

Tridham (TD) is a polyherbal formulation of three ingredients, *Terminalia chebula* seed coat, *Elaeocarpus ganitrus* fruits, and *Prosopis cineraria* leaves in equal proportion routinely used by the traditional Indian medicinal practitioners in the treatment of cancer. *Terminalia chebula* is a multipurpose herbal with excellent antibacterial [21], antifungal, antiviral [22], anticarcinogenic [23], antianaphylactic [24], antidiabetic [25], and antioxidant [26] properties. *Elaeocarpus ganitrus* commonly known as Rudraksha in India is grown in Assam and the Himalayan regions of India for medicinal properties [27]. It has excellent free radical scavenging effect in rats [28]. Besides, it is reported to exhibit multifarious pharmacological activities that include anti-inflammatory [29], analgesic, sedative [30], antidepressant, antiasthmatic [31], hypoglycemic [32], antihypertensive [33], and smooth muscle relaxant [34]. *Prosopis cineraria *(Syn.* P. spicigera* L.) possesses antibacterial, antifungal, antiviral, and several other pharmacological properties [35]. The smoke of the leaves is considered good for eye ailments. Leaves of *P. cineraria* are rich in phytochemical constituents like alkaloids, namely, spicigerine; steroids, namely, campesterol, cholesterol, sitosterol, stigmasterol; alcohols namely octacosanol and triacontan-1-ol; and alkane hentriacontane [36].

The qualitative chemical exposition studies (data not shown) on TD showed the presence of various beneficial phytochemicals such as flavonoids, tannins, alkaloids, and polyphenols. A known compound 3,4,5-trihydroxybenzoic acid (Gallic acid) has been isolated through column chromatography and elucidated by a series of experiments, involving NMR, IR, MS, and single-crystal X-ray crystallography (XRD)(unpublished data). The isolated Gallic acid is a well known polyhydroxyphenolic compound that can be found in various natural products.

Despite innumerable studies indicating the utility of individual medicinal herbs in the treatment of various clinical manifestations, their application in cancer management is still in its initial phase. With innumerable clinically relevant active principles enriched in each component, TD is a herbal preparation with promise in combating the progression of cancer. The present study is aimed at evaluating the antioxidant potential of TD in overcoming oxidative damages associated with AFB_1_-instigated HCC in male Wistar albino rats.

## 2. Materials and Methods

### 2.1. Materials

#### 2.1.1. Animals and Diet

Male albino rats of Wistar strain, 8–10 weeks of age (120–150 g), were used in this study. The animals were obtained from Central Animal House Facility, Dr. ALM PG IBMS, University of Madras, Taramani, Chennai, India. The animals were housed in polypropylene cages under a controlled environment with 12 ± 1 h light/dark cycles and a temperature between 27 and 37°C and were fed with standard pellet diet (Gold Mohor rat feed, M/s. Hindustan Lever Ltd., Mumbai) and water *ad libitum*. All experiments involving animals were conducted according to NIH guidelines, after obtaining approval from the institute's animal ethical committee (*IAEC *no.* 06/012/08*).

#### 2.1.2. Chemicals

AFB_1_ was procured from Sigma Chemical Co., St. Louis, MO. It was dissolved in dimethyl sulphoxide (DMSO) immediately before administration. All other chemicals used were of highest purity and analytical grade.

#### 2.1.3. TD Preparation and Dose Determination

TD drug is a combination of *Terminalia chebula* seed coats (family: Combretaceae), dry seeds of *Elaeocarpus ganitrus* (Syn. *E. sphaericus*) (family: Elaeocarpaceae) and *Prosopis cineraria* leaves (Syn. *P. spicigera* L.) (family: Leguminosae). The three ingredients were collected and given to the Department of Centre for Advance Study (CAS) in Botany, University of Madras, Guindy Campus, Chennai, India for botanical authentication. The assigned herbarium numbers are CASBH-16: *Terminalia chebula,* CASBH-17: *Elaeocarpus ganitrus,* CASBH-18: *Prosopis cineraria. *The Ingredients were washed, air-dried in shade, and then finely ground. The components were then mixed in equal proportions on weight basis to get TD mixture. The extract of TD was prepared in 3 : 1 (v/w) ratio by adding 30 mL of water to 10 grams of combined TD and mixed well. The mixture was mixed by using a shaker for 12 hours. The mixture was subsequently filtered using filter paper, and the clear filtrate (aqueous extract) was collected in a beaker. The filtrate was then lyophilized under vacuum pressure to yield a powder. The lyophilized extract was stored in airtight containers in a dry dark place.

The dose of TD used, that is, 300 mg/kg body weight/day, was decided upon after carrying out a dose-dependent study. Acute toxicity studies showed no mortality and sub-acute toxicity studies showed no significant changes in histopathological, hematological, and biochemical parameters. Based on these studies, we carried out a dose-dependent study at doses of 50, 100, 200, 300, 400 mg/kg body weight/day. The results of this study revealed that the lowest possible effective dose was 300 mg/kg body weight as evidenced by histopathological observations, a significant (*P* < 0.05) increase in body weight and a significant (*P* < 0.05) reduction in the levels of marker enzymes in serum and liver (Unpublished data). The duration of treatment, that is, 45 days is the duration used by Siddha (a traditional Indian system of medicine) physicians for treating HCC patients utilizing the formulation TD. Thus the optimum dose of TD was found to be 300 mg/kg body weight/day for 45 days, and this dose was used for all the subsequent experiments.

#### 2.1.4. Experimental Design

Animals were divided into following four groups of six animals each. AFB_1_ was freshly prepared by dissolving in dimethyl sulphoxide (DMSO) before administration.

Group I. Normal control animals
Group II. HCC was induced in these animals by a single intra-peritoneal dose of AFB_1_ (2 mg/kg body weight) [37].
Group III. HCC-induced animals (as in Group II) were administered with the drug, TD (300 mg/kg body weight/day) orally for 45 days.
Group IV. Drug control animals received the same dosage of TD as in Group III animals.

On the completion of the experimental period, animals were sacrificed by cervical decapitation between 8:00 and 10:00 h to avoid any possible rhythmic variations in the antioxidant enzyme level. Blood was collected. Liver and kidney were simultaneously removed, washed with ice-cold saline. The organs were weighed, and one portion of each of these organs was fixed in 10% formalin for histopathological examinations. 10% homogenate was prepared with fresh tissue in 0.01 M Tris-HCl buffer (pH 7.4). The resultant supernatant was then used for biochemical assays.

### 2.2. Methods

#### 2.2.1. Biochemical Investigations

Total protein was estimated by the method of Lowry et al. [38] using bovine serum albumin (BSA) as the standard. LPO was measured by the method of Devesagayam and Tarachand [39]. The TBARs were estimated as per the spectrophotometric method described by Ohkawa et al. [40]. PCOs were measured by the method of Reznick and Packer [41]. SOD was assayed by the method of Marklund and Marklund [42]. CAT was assayed by the method of Sinha [43]. Glutathione peroxidase (GPx) was assayed by the method of Rotruck et al. [44]. GSH was determined by the method of Moron et al. [45]. Vitamin E was estimated by the method of Quaife and Dju [46]. Vitamin C was estimated by the method of Omaye et al. [47].

#### 2.2.2. Statistical Analysis

The values are expressed as mean ± SD for six rats in each group. Statistically significant differences between the groups were calculated using one-way analysis of variance (ANOVA) employing statistical package for social sciences (SPSS). Values of *P* < 0.05 were considered to be significant.

#### 2.2.3. Gross Morphology and Histopathological Studies

Portions of tissues were then fixed in 10% neutral buffered formalin, routinely processed, and embedded in paraffin wax. Consecutive sections were cut at a thickness of 3-4 *μ*m and subsequently stained with hematoxylin and eosin [48].

## 3. Results

### 3.1. Macromolecular Damage

Tables 1 and 2 depict the levels of LPO, TBARs, and PCO in liver and kidney of control and experimental animals. There was a significant increase in LPO, TBARs, and PCO in the HCC-induced (Group II) animals. The levels of these parameters were restored to near-normal levels on treatment with TD in Group III animals.

### 3.2. Antioxidants

**Figure 1:**
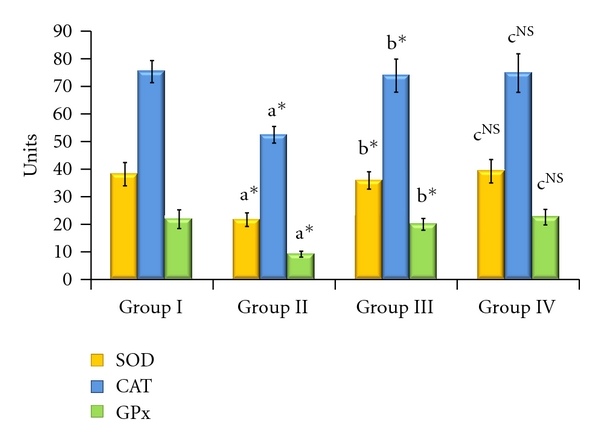
Effect of TD on enzymic antioxidants in serum of control and experimental animals. Values are expressed as mean ± SD for six animals. Comparisons are made between “a” Group II versus Group I, “b” Group III versus Group II, “c” Group IV versus Group I. The symbol *represents the statistical significance at *P* < 0.05 NS: nonsignificant. SOD: U/min/mg protein, CAT: *μ*moles of H_2_O_2_ consumed/min/mg protein, GPx: *μ*moles of GSH oxidized/min/mg protein.

**Figure 2:**
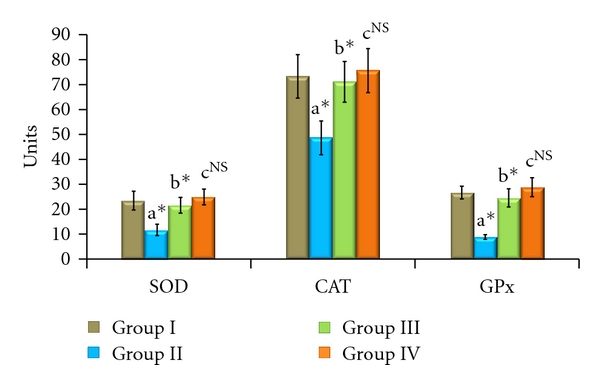
Effect of TD on enzymic antioxidants in liver of control and experimental animals. Values are expressed as mean ± SD for six animals. Comparisons are made between “a” Group II versus Group I, “b” Group III versus Group II, “c” Group IV versus Group I. The symbol *represents the statistical significance at *P* < 0.05. NS: nonsignificant. SOD: U/min/mg protein, CAT: *μ*moles of H_2_O_2_ consumed/min/mg protein, GPx: *μ*moles of GSH oxidized/min/mg protein.

**Figure 3:**
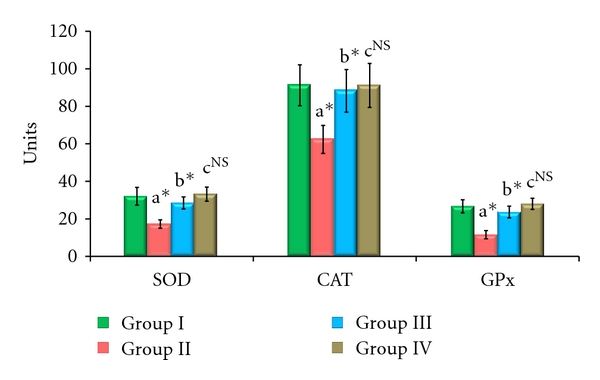
Effect of TD on enzymic antioxidants in kidney of control and experimental animals. Values are expressed as mean ± SD for six animals. Comparisons are made between “a” Group II versus Group I, “b” Group III versus Group II, “c” Group IV versus Group I. The symbol *represents the statistical significance at *P* < 0.05. NS: nonsignificant. SOD: U/min/mg protein, CAT: *μ*moles of H_2_O_2_ consumed/min/mg protein, GPx: *μ*moles of GSH oxidized/min/mg protein.

The enzymic antioxidant activities of SOD, CAT, and GPx in the serum, liver, and kidney are shown in Figures 1, 2, and 3, respectively. From these figures, it is evident that the activities of enzymic antioxidants were significantly decreased in AFB_1_-induced animals (Group II). HCC bearing animals receiving TD treatment (Group III) attained a near-normal level of enzymic antioxidant activities. The levels of antioxidant enzymes remained constant without showing any significant change in Group IV drug control animals.

The levels of nonenzymic antioxidants like vitamin C, vitamin E, GSH, total thiols (TTs), and nonprotein thiols (NPT) in the serum, liver, and kidney are depicted in Tables 3, 4, and 5. Similar to enzymic antioxidant status, the levels of non-enzymic antioxidants were also decreased significantly in HCC animals (Group II), which were reverted to near-normal levels after treatment with TD in Group III animals. No significant variation in the level of non-enzymic antioxidants was noted in Group IV drug control animals.

### 3.3. Gross Morphology and Histopathology

**Figure 4:**
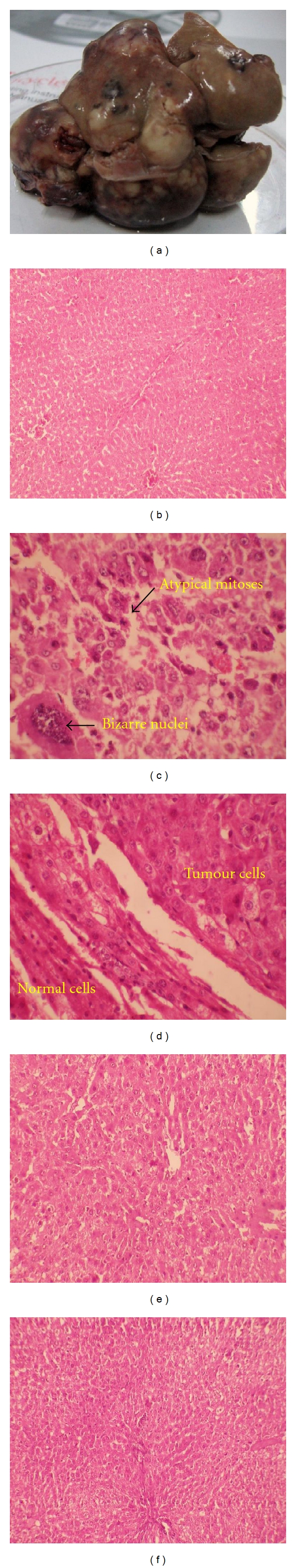
Effect of TD on gross morphology and histopathology in liver of control and experimental animals. (a) Gross morphology of Group II animals (AFB_1_ induced). (b) Liver tissue of Group I control animals (10X). (c) Liver tissue of Group II (AFB_1_ induced) animals (40X). (d) Liver tissue of Group II (AFB_1_ induced) animals (40X). (e) Liver tissue of Group III (AFB_1_ induced + TD treated) animals (10X). (f) Liver tissue of Group IV (TD treated) animals (10X).

Grossly, liver tissue of Group II animals (AFB_1_ induced) showed whitish nodular tumours of varying sizes in the liver (Figure 4(a)).

 Microscopically, Group I control animals showed liver tissue with normal histology (Figure 4(b)). The liver of Group II (AFB_1_ induced) animals was infiltrated by hyperchromatic pleomorphic tumour cells arranged in a trabecular pattern in some areas and in sheets and nests in other areas. Numerous mitotic figures including atypical mitoses were seen. Some of the tumour cells showed markedly enlarged, bizarre nuclei (Figure 4(c)). The adjacent normal liver cells showed nodularity and dysplastic features (Figure 4(d)). Group III (AFB_1_ induced TD treated) liver showed normal histology. There was no evidence of tumour (Figure 4(e)). Group V (TD alone) liver showed normal morphology and architecture (Figure 4(f)).

## 4. Discussion

LPO is a well-recognized preliminary event of oxidative damage of plasma membrane and initiation of carcinogenesis [49]. The formation of the metabolite, AFB_1_-8,9-epoxide, causes membrane damage through lipid peroxidation and subsequent covalent binding to DNA to form AFB_1_-DNA adducts. These are critical steps leading to hepatocarcinogenesis [50]. There was a significant reduction in the level of lipid peroxides upon treatment with TD in tumour-bearing animals which have been mainly attributed to the components of TD. Aqueous extract of *T*.* chebula* at a concentration of 15 *μ*g/mL shows 50% inhibition in LPO activity [51]. A report by Suchalatha and Devi [52] strongly indicate a protective role of *T. chebula* against membrane damage by prevention of peroxide radical formation and MDA formation in isoproterenol-induced oxidative stress in rats. *E. ganitrus* acts as a potent iron chelator with 76.70% inhibition at a concentration of 500 *μ*g/mL. Metal chelating agents reduce the concentration of catalyzing transition metals by forming sigma bonds and reducing the redox potential, thereby stabilizing the oxidized form of the metal ion [53]. Malon dialdehyde acts as an important contributor to the increase in protein carbonyl content observed during the oxidation of protein/polyunsaturated fatty acid mixtures [54]. Dietary antioxidant and polyphenols act against ROS thereby indirectly reducing the PCO content [55]. The inhibitory effects of PCO by TD may be attributed to the presence of various bioactive components such as flavonoids, alkaloids, and polyphenols that act as antioxidants by scavenging chain-propagating, reactive free radicals generated by AFB_1._

The reducing capacity of a compound serves as a significant indicator of its potential antioxidant activity. Levels of enzymic antioxidants were significantly decreased in HCC-bearing animals as these are consumed for reducing prooxidants. Supplementation of TD to tumour-bearing animals restored the level of different antioxidants, effectively. Flavonoids have been shown to act as scavengers of various oxidizing species, such as hydroxyl radicals, peroxy radicals, or superoxide anions, due to the presence of a catechol group in the B-ring and the 2,3 double bond in conjunction with the 4-carbonyl group as well as the 3- and 5-hydroxyl groups [56, 57]. Flavonoids have been proved to be potent inhibitors of enhanced spontaneous production of both MDA and conjugated dienes [58]. Antioxidant activity of TD results mainly from ellagic acid, 2,4-chebulyl-b-D-glucopyranose, chebulinic acid, casuarinin, chelani, and 1,6-di-*O*-galloyl-b-D-glucose which have been reported to be active constituents in *T. chebula* fruits [23, 26]. Aqueous extract of fruit of *T. chebula *should strengthen antioxidant properties, presenting *tert*-butyl hydroperoxide- (*t*-BHP-) induced oxidative injury observed in cultured rat primary hepatocytes and rat liver [59]. Glutathione peroxidase (GPx) is considered as a major defence against peroxides, superoxide anion, and hydrogen peroxide and assumes an important role in detoxifying lipid and hydrogen peroxide with the concomitant oxidation of glutathione [60]. Phytochemicals present in T*. chebula* may contribute to restoration of GPx activity by TD treatment observed in this study. These antioxidant activities of TD are based on hydrogen donation abilities and chelating metal ions.

 GSH plays a major role in the detoxification of xenobiotic compounds [61]. The high levels of flavonoids and phenolic compounds present in components of TD have been reported to have the capacity to increase GSH levels, modifying its redox rate and actively, participating in the eliminating of AFB_1_ metabolite [62]. Sharma et al. [63] have reported that phenolic compounds are found to be inducers of GSH. Gallic acid, a polyphenol, is one of the hepatoprotective active principles isolated from TD that may augment GSH levels. Vitamin C is one of the most effective biological antioxidants, and it has been shown that vitamin C supplementation can reduce risks of diseases associated with oxidative stress, such as cancer [64]. Among the factors modifying oxidative stress, there is strong interest in the antioxidant vitamins E and C, the intake of which can be easily and safely controlled through the diet. Vitamin C protects cells mainly against ROS such as superoxide anion radical, hydroxyl oxygen radical, hydrogen peroxide, and singlet oxygen [65]. It is the most significant antioxidant that can protect against carcinogenesis and tumour growth. A decreased level of vitamin E content might be due to the excessive utilization of this antioxidant for quenching the enormous quantity of free radicals produced in these conditions. According to Hazra et al. [66] *T. chebula* is known for its natural antioxidant property due to its content of vitamins C. Thus, vitamin C and E could act synergistically in scavenging a wide variety of ROS. Thiols are water-soluble antioxidants linked to membrane proteins and are essential for the antioxidant system. AFB_1_ administration causes the reduction of thiol levels [67]. Both total and nonprotein thiols were decreased in AFB_1_-induced HCC conditions. Due to the antioxidant and free radical quenching nature of TD, the thiol levels were resumed to near normal in drug-treated animals. TD acts as potential antioxidant agent by exerting its activity at various levels to reduce AFB_1_-induced oxidative stress and effectively quenches free radicals, reduces its formation, and increases the activity of different antioxidant enzymes. Thus TD has a hepatoprotective effect as demonstrated by enhanced activity of antioxidant enzymes. The redeeming action of TD in tumour-induced rats is most probably due to additive and synergistic effect of individual components.

Compounds such as tannins are reported to be responsible for retrieval of vitamin C activity [68], whereas trigalloyl glucose and ellagic acid [69] present in TD might strengthen the soothing activity of the drug. We have evaluated the antioxidant potential of TD by studying the combined effects of three ingredients derived from *T. chebula*,* E. ganitrus *and* P. cineraria* in AFB_1_-induced liver cancer. The active principles present in each component render TD a potent antioxidant property. The therapeutic effect of TD in tumour-induced rats is most probably due to the synergistic action of the constituents such as flavonoids, alkaloids and tannins. *T. chebula *contains the flavonoids, gallic acid, 1,2,3,4,6-penta-*O*-galloyl-b-D-glucopyranose, chebulagic acid and chebulinic acid as well as vitamin C [70]. A report from Ray et al. [71] reveals the presence of alkaloids, glycosides, steroids, and flavonoids in *E. ganitrus. *Malik and Kalidhar [36] have reported the presence of tannins, alkaloids and steroids in *Prosopis cineraria* leaves. Thus TD has been found to exhibit hepatoprotective and anticarcinogenic effect as demonstrated by increased activity of antioxidant enzymes and total regression of tumour seen on histopathological examination of the liver. In summary, the present study provides the evidence that TD has therapeutic effect in AFB_1_-induced HCC. TD also reverses the free radical damage brought about by administration of AFB_1_ by enhancing the enzymatic and non enzymatic antioxidant defense mechanisms.

## 5. Conclusion

The protection by TD against oxidative stress in AFB_1_-mediated HCC might has occurred through multiple actions, which include prevention of LPO and stabilization of antioxidant defense mechanism. These factors protect cells from ROS damage in AFB_1_-induced HCC, as TD abolishes the causative factors of liver injury and tumour markers by decreasing LPO, the possible mechanism of AFB_1_ induction. The strong antioxidant and therapeutic effect of TD *in vivo *might be due to the spectrum of synergistically active phytomolecules present in TD. Elucidation of the exact mechanism of action of the phytotherapeutic effect of TD in AFB_1_-induced HCC necessitates further studies.

## Figures and Tables

**Table 1:** Effect of TD on indicators of macromolecular damage in liver of control and experimental animals.

Parameters	Group I (control)	Group II (AFB_1_ induced)	Group III (AFB_1_ induced + TD)	Group IV (TD alone)
Lipid peroxides (LPO)	51.64 ± 5.62	79.41 ± 9.26^a∗^	53.15 ± 6.14^b∗^	50.44 ± 5.02^cNS^
Thiobarbituric acid reactive substances (TBARSs)	3.89 ± 0.41	7.74 ± 0.73^a∗^	4.25 ± 0.49^b∗^	3.93 ± 0.38^cNS^
Protein carbonyls (PCOs)	6.13 ± 0.57	14.75 ± 1.78^a∗^	8.31 ± 0.89^b∗^	5.96 ± 0.64^cNS^

*Units*. LPO: nmoles of MDA formed/mg protein, TBARS: nmoles/100 g tissue, protein carbonyl: nmoles of DNPH formed/min/mg protein. Values are expressed as mean ± SD for six animals. Comparisons are made between “a” Group II versus Group I, “b” Group III versus Group II, “c” Group IV versus Group I. The symbol *represents the statistical significance at *P* < 0.05. NS: nonsignificant.

**Table 2:** Effect TD on indicators of macromolecular damage in kidney of control and experimental animals.

Parameters	Group I (control)	Group II (AFB_1_ induced)	Group III (AFB_1_ induced + TD)	Group V (TD alone)
Lipid peroxides (LPO)	46.87 ± 3.62	68.17 ± 6.38^a∗^	48.53 ± 5.21^b∗^	45.87 ± 4.76^cNS^
Thiobarbituric acid reactive substances (TBARSs)	3.13 ± 0.37	7.39 ± 0.69^a∗^	3.95 ± 0.44^b∗^	3.18 ± 0.35^cNS^
Protein carbonyls (PCOs)	5.53 ± 0.59	11.61 ± 1.31^a∗^	6.14 ± 0.69^b∗^	5.56 ± 0.61^cNS^

*Units*: LPO: nmoles of MDA formed/mg protein, TBARS: nmoles/100 g tissue, protein carbonyl: nmoles of DNPH formed/min/mg protein. Values are expressed as mean ± SD for six animals. Comparisons are made between “a” Group II versus Group I, “b” Group III versus Group II, “c” Group IV versus Group I. The symbol *represents the statistical significance at *P* < 0.05. NS: nonsignificant.

**Table 3:** Effect of TD on non enzymic antioxidants and thiols in serum of control and experimental animals.

Parameters	Group I (control)	Group II (AFB_1_ induced)	Group III (AFB_1_ induced + TD)	Group IV (TD alone)
Vitamin C	2.83 ± 0.29	0.99 ± 0.01^a∗^	2.87 ± 0.28^b∗^	2.85 ± 0.31^cNS^
Vitamin E	2.43 ± 0.28	1.09 ± 0.19^a∗^	2.38 ± 0.27^b∗^	2.45 ± 0.26^cNS^
Total thiols (TSHs)	5.16 ± 0.59	3.07 ± 0.39^a∗^	5.09 ± 0.51^b∗^	5.17 ± 0.53^cNS^
Non protein thiols (NPSHs)	4.89 ± 0.51	1.69 ± 0.19^a∗^	4.66 ± 0.51^b∗^	4.97 ± 0.52^cNS^
Reduced glutathione (GSH)	24.68 ± 2.92	9.45 ± 1.67^a∗^	23.35 ± 3.16^b∗^	24.63 ± 2.67^cNS^

*Units*: GSH mg/100 g tissue, Vitamins C and E mg/dL tissue, TSH and NPSH: *μ*g/mg protein. Values are expressed as mean ± SD for six animals. Comparisons are made between “a” Group II versus Group I, “b” Group III versus Group II, “c” Group IV versus Group I. The symbol *represents the statistical significance at *P* < 0.05. NS: nonsignificant.

**Table 4:** Effect of TD on nonenzymic antioxidants and thiols in liver of control and experimental animals.

Parameters	Group I (control)	Group II (AFB_1_ induced)	Group III (AFB_1_ induced + TD)	Group IV (TD alone)
Vitamin C	3.93 ± 0.49	1.95 ± 0.27^a∗^	3.69 ± 0.43^b∗^	4.11 ± 0.51^cNS^
Vitamin E	2.54 ± 0.35	1.36 ± 0.17^a∗^	2.38 ± 0.28^b∗^	2.45 ± 0.31^cNS^
Total thiols (TSHs)	9.42 ± 1.13	4.68 ± 0.56^a∗^	8.81 ± 1.14^b∗^	9.31 ± 1.11^cNS^
Non protein thiols (NPSHs)	5.58 ± 0.66	2.15 ± 0.25^a∗^	5.31 ± 0.68^b∗^	5.44 ± 0.65^cNS^
Reduced glutathione (GSH)	32.31 ± 4.21	18.34 ± 2.77^a∗^	29.73 ± 4.75^b∗^	33.83 ± 4.17^cNS^

*Units*: GSH mg/100 g tissue, Vitamin C and E mg/g wet tissue, TSH and NPSH: *μ*g/mg protein. Values are expressed as mean ± SD for six animals. Comparisons are made between “a” Group II versus Group I, “b” Group III versus Group II, “c” Group IV versus Group I. The symbol *represents the statistical significance at *P* < 0.05. NS: nonsignificant.

**Table 5:** Effect of TD on nonenzymic antioxidants and thiols in kidney of control and experimental animals.

Parameters	Group I (control)	Group II (AFB_1 _induced)	Group III (AFB_1_ induced + TD)	Group IV (TD alone)
Vitamin C	2.94 ± 0.38	1.52 ± 0.22^a∗^	2.78 ± 0.35^b∗^	2.95 ± 0.37^cNS^
Vitamin E	2.85 ± 0.29	1.12 ± 0.17^a∗^	2.42 ± 0.27^b∗^	2.91 ± 0.31^cNS^
Total thiols (TSH)	7.17 ± 1.14	2.88 ± 0.33^a∗^	6.85 ± 0.78^b∗^	7.79 ± 0.81^cNS^
Non protein thiols (NPSH)	4.75 ± 0.61	2.68 ± 0.34^a∗^	4.31 ± 0.51^b∗^	4.68 ± 0.56^cNS^
Reduced glutathione (GSH)	27.04 ± 2.97	13.54 ± 2.21^a∗^	25.51 ± 2.98^b∗^	27.98 ± 3.23^cNS^

Units-GSH-mg/100 g tissue, Vitamin C and E-mg/g wet tissue, TSH and NPSH: *μ*g/mg protein. Values are expressed as mean ± SD for six animals. Comparisons are made between “a” Group II versus Group I, “b” Group III versus Group II, “c” Group IV versus Group I. The symbol *represents the statistical significance at *P* < 0.05. NS: nonsignificant.
